# Contrast-enhanced mammography (CEM) *versus* MRI for breast cancer staging: detection of additional malignant lesions not seen on conventional imaging

**DOI:** 10.1186/s41747-022-00318-5

**Published:** 2023-02-13

**Authors:** Donna B. Taylor, Sally Burrows, Christobel M. Saunders, Paul M. Parizel, Angela Ives

**Affiliations:** 1 Department of Diagnostic and Interventional Radiology, Royal Perth Hospital, Wellington Street, Perth, 6000 WA Australia; 2 Medical School, The University of Western Australia (M570), 35 Stirling Highway, Perth, Australia; 3 Department of Surgery, Royal Melbourne Hospital, 300 Grattan Street, Parkville, VIC Australia

**Keywords:** Biopsy (needle), Breast neoplasms, Mammography, Magnetic resonance imaging, Neoplasm staging

## Abstract

**Background:**

Contrast-enhanced mammography (CEM) is more available than MRI for breast cancer staging but may not be as sensitive in assessing disease extent. We compared CEM and MRI in this setting.

**Methods:**

Fifty-nine women with invasive breast cancer underwent preoperative CEM and MRI. Independent pairs of radiologists read CEM studies (after reviewing a 9-case set prior to study commencement) and MRI studies (with between 5 and 25 years of experience in breast imaging). Additional lesions were assigned National Breast Cancer Centre (NBCC) scores. Positive lesions (graded NBCC ≥ 3) likely to influence surgical management underwent ultrasound and/or needle biopsy. True-positive lesions were positive on imaging and pathology (invasive or *in situ*). False-positive lesions were positive on imaging but negative on pathology (high-risk or benign) or follow-up. False-negative lesions were negative on imaging (NBCC < 3 or not identified) but positive on pathology.

**Results:**

The 59 women had 68 biopsy-proven malignant lesions detected on mammography/ultrasound, of which MRI demonstrated 66 (97%) and CEM 67 (99%) (*p* = 1.000). Forty-one additional lesions were detected in 29 patients: six of 41 (15%) on CEM only, 23/41 (56%) on MRI only, 12/41 (29%) on both; CEM detected 1/6 and MRI 6/6 malignant additional lesions (*p* = 0.063), with a positive predictive value (PPV) of 1/13 (8%) and 6/26 (23%) (*p* = 0.276).

**Conclusions:**

While MRI and CEM were both highly sensitive for lesions detected at mammography/ultrasound, CEM may not be as sensitive as MRI in detecting additional otherwise occult foci of malignancy.

**Trial registration:**

Australian and New Zealand Clinical Trials Registry: ACTRN 12613000684729

**Supplementary Information:**

The online version contains supplementary material available at 10.1186/s41747-022-00318-5.

## Key points

• Contrast-enhanced mammography (CEM) detected fewer additional lesions than magnetic resonance imaging (MRI), with 29% false positives.
• CEM failed to detect five of six additional cancers: two invasive ductal cancers (5 and 7 mm), two ductal carcinoma *in situ* (2 and 30 mm), and an invasive lobular cancer metastatic intramammary lymph node (10 mm).
• Reasons for CEM false negatives included lesion out of field of view (*n* = 1) and superimposition and/or non-enhancement (*n *= 4).
• Despite producing 49% false positives, MRI detected all six additional cancers.
• CEM may not be as sensitive as MRI for breast cancer staging.

## Background

Accurate assessment of the local extent of breast cancer is essential to plan treatment. While knowing the size of the index malignant lesion preoperatively may help the surgeon to obtain clear pathological margins, and reduce rates of re-excision [1], detection of additional malignant lesions in the same quadrant (multifocal) or different quadrants (multicentric) quadrants or in the contralateral breast is arguably even more important.

Breast cancer patients with multifocal or multicentric disease have higher risk of lymph node metastasis and poorer prognosis [2]. Relapses after conservative surgery are frequently due to undetected malignant foci [3]. Preoperative identification of multicentric disease usually precludes attempted breast conserving surgery and presents a contraindication to either omission of whole breast radiotherapy (RT) or use of targeted RT. Synchronous detection of contralateral breast cancer enables contemporaneous bilateral treatment, avoiding the additional stresses associated with metachronous lesion detection, investigation, and treatment.

MRI has long been considered the most sensitive approach for determining breast cancer extent [4–6]. However, suboptimal specificity and positive predictive value, limited availability, and patient tolerance as well as high cost have encouraged the development of alternative techniques. CEM is a relatively new technique that produces images of both high-resolution morphology and functional information about tissue neoangiogenesis. Mammograms are obtained with low-energy and high-energy x-ray exposures following intravenous injection of iodinated contrast. The low-energy images are equivalent to a standard mammogram [7]. Logarithmic subtraction of low-energy and high-energy image data gives recombined images showing areas of iodine uptake as bright, while anatomic noise is suppressed. CEM appears to be cheaper, easier to access, and quicker to perform and interpret than MRI [8, 9]. Women who have previously undergone both tests express a clear preference for CEM [10]. Recent studies comparing CEM with MRI have reported CEM to have similar sensitivity [11–13] for detection of malignant disease, with fewer false-positive findings [14].

The aim of this paper is to compare the ability of CEM and MRI to detect additional malignant lesions not evident on routine clinical examination and conventional breast imaging in women with invasive breast cancer.

## Methods

This prospective study (Australian and New Zealand Clinical Trials Registry: ACTRN 12613000684729) was conducted at two tertiary referral hospitals between June 2013 and October 2015. The study was approved by the Institutional Human Research and Ethics Committee, and compliant with the National Health and Medical Research Council Statement on Ethical Conduct in Human Research. Written informed consent was obtained from all participants.

### Study sample

Women over the age of 21 years with at least one biopsy-proven invasive breast cancer who were fit for surgery were invited to participate. Patients with renal failure, history of allergy to iodinated or gadolinium contrast agent, diabetes treated with metformin, pregnancy or lactation, contraindications to MRI, or breast implants were excluded.

### Imaging protocols for detection and interpretation of additional lesions and of background parenchymal enhancement

Conventional imaging (which may have included digital mammography, coned compression, magnification views, tomosynthesis, and breast ultrasound) was performed at outside practices or in our clinic. Following study enrolment, each participant underwent both MRI and CEM (with order determined by appointment availability) within 15 days of initial diagnosis. The protocols used for the acquisition of CEM and MRI images have been previously described in detail [15]. In summary, dual-energy CEM was performed using a Senographe DS system (GE Healthcare Australia Pty Ltd, Rydalmere, NSW, Australia). Craniocaudal and mediolateral oblique views of both breasts (CC unaffected breast, CC affected breast, MLO unaffected breast, MLO affected breast) were obtained 2 min after intravenous (IV) injection of 1.5 mL per kg of non-ionic contrast (Iohexol) containing 350 mg iodine per mL at 3 mL per second. All images were acquired within 9 min from injection.

MRI was performed using a 1.5-T Siemens Sonata Maestro Class machine (Siemens Healthcare, Erlangen, Germany), and a Siemens 4-channel breast coil. Unenhanced axial T2- and T1-weighted images were followed by multiple T1-weighted images commencing 90 s after intravenous injection of 0.1 mmol/kg of gadolinium-diethylenetriamine pentaacetic acid (Magnevist, Bayer Healthcare: Whippany, NJ, USA) at 3 mL/s.

Each study was independently read on Agfa Picture Archiving and Communications System workstations (Agfa-Gevaert NV Mortsel, Belgium) by two sub-specialist breast radiologists, and a consensus report issued. In the event of disagreement, third-reader arbitration was used. The radiologists who read the MRIs had between 5 and 25 years of experience in breast imaging while the radiologists who read the CEM studies had reviewed a multimodality training set of nine cases (a mixture of six unifocal and multifocal invasive cancers, with and without associated DCIS component, and three with benign findings) prior to study commencement.

Background parenchymal enhancement on both CEM and MRI was assessed according to the MRI Breast Imaging Reporting and Data System (BI-RADS) classification [16] and dichotomised for purposes of analysis into minimal-mild and moderate-marked.

### Definition and classification of additional lesions

**Fig. 1:**
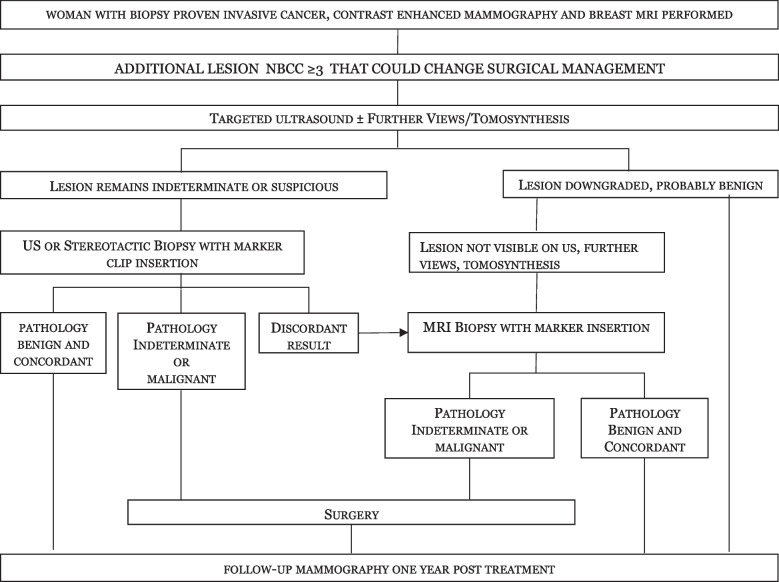
Assessment pathway for additional lesions. *CEM* Contrast-enhanced mammography, *FV* Further mammographic views, *NBCC* National Breast Cancer Center, *Tomo* Tomosynthesis, *US* Ultrasound

Additional lesions were those identified with CEM and/or MRI, which had not been previously detected with routine clinical examination and conventional breast imaging (as defined above). Additional lesions were assigned an NBCC grade of between 1 and 5 according to the radiologists’ level of concern (1 = benign; 2 = probably benign; 3 = indeterminate/equivocal; 4 = suspicious; 5 = malignant) [17]. Lesions graded NBCC 3 and above considered likely to influence the surgical plan underwent work-up with further mammographic views/tomosynthesis and/or targeted ultrasound as shown in Fig. 1. Lesions that remained indeterminate or suspicious underwent needle biopsy with insertion of a tissue marker if core needle biopsy was performed. Lesions with concordant benign imaging findings were downgraded.

Based on the CEM and/or MRI imaging findings, lesions classified as NBCC category 1 or 2 were considered negative, and those classified NBCC 3 and above were called positive. A lesion not detected on one or other modality was considered as negative for that modality.

### Reference standard

The reference standard used to verify the positive or negative status of additional lesions were pathology findings (at needle biopsy or excision) or follow-up imaging. As per standard of care (in absence of bilateral mastectomy), all participants underwent mammographic follow-up at 12 months. A 6- or 12-month post-surgical CEM or MRI exam was allowed for follow-up of additional lesions graded NBCC 3 and above that had been downgraded following work-up.

The study pathologist was given a diagram for each patient (see Supplementary material), showing the location of all imaging detected lesions usually prior to sectioning the excised tissue, to facilitate blocking of regions of interest for histological review. Specimen radiographs were also reviewed. Details regarding the pathology processing techniques are provided in the Supplementary material.

The presence or absence of a pathological correlate for each image-detected lesion was recorded. Retrospective review of each patient’s pathology and imaging findings by the first author and a study pathologist for lesion concordance was also undertaken.

Lesions shown to be invasive carcinoma, ductal carcinoma *in situ* (DCIS), or pleomorphic lobular carcinoma *in situ* were classified as positive. Benign findings and lesions of indeterminate pathological significance (such as atypical ductal hyperplasia, lobular neoplasia, papillomas, flat epithelial atypia, and radial scar) were classified as negative. Lesions that were not sampled that had been downgraded following further imaging work-up and/or shown stability or resolution on follow-up imaging for at least 12 months were also considered negative.

True positive lesions were those called positive on imaging that had positive pathology correlate. False-positive lesions were those called positive on CEM or MRI with a concordant negative reference standard. False-negative lesions were those that were positive on pathology without suspicious findings on CEM or MRI. As outlined by Moskowitz et al. [18], true-negative findings cannot be determined in a lesion-level analysis.

### Statistical analysis

An exact binomial test was used to compare modality performance in detecting each of the index or additional lesions identified by either modality. Patient demographics, breast imaging, and the pathology findings were reported using descriptive statistics and the numbers of true- and false-positive test results were tabulated.

Sensitivity was calculated despite the presence of verification bias, for comparison with other studies (also subject to verification bias) [18]. Specificity and negative predictive values were not calculated due to the inability to determine the number of true negatives in a lesion-level analysis. An exact binomial test was used to compare the CEM and MRI sensitivity estimates for the detection of additional malignant lesions. Positive predictive values were compared using a random effects logistic regression analysis.

Analyses were undertaken using SAS version 9.4 (SAS Institute Inc. Cary, NC, USA) and Stata 17 (StataCorp. 2021. Stata Statistical Software: Release 17. College Station, TX, USA: StataCorp LL).

## Results

Final pathology and follow-up results were available for 59 women who had completed both MRI and CEM and who had not undergone neoadjuvant systemic treatment. Both tests were completed within 25 days from study enrolment, with 75% completed within 12 days. The age of the patients was 56 ± 11 years (mean ± standard deviation), ranging from 35 to 77 years. Most cases were asymptomatic (lesions detected on screening mammography); however, 11 patients had palpable masses related to index cancers. Two patients had previous history of breast cancer (ipsilateral in one and contralateral in another). Four patients had a family history of breast cancer and one of ovarian cancer. No patients had a known gene mutation.

### Background parenchymal enhancement (BPE)

BPE was assessed as minimal-mild by both MRI and CEM in 38 women and moderate-marked in 12. Of the remaining nine women, MRI assessed BPE to be more substantial than CEM in six (*p* = 0.508).

### Malignant lesions identified by conventional imaging before CEM and MRI

The 59 women had 68 biopsy-proven malignant lesions that had been detected on mammography and/or ultrasound, prior to MRI and CEM. MRI demonstrated 66/68 (97%) of these malignant lesions and CEM 67/68 (99%) (*p* = 1.000.

### Additional lesions only detected by MRI and CEM

There were 41 additional lesions detected in 29/59 patients (49%): 18 women had one lesion, 10 women had two lesions, and one woman had three additional lesions (Table 1).

**Table 1:** Characteristics of the 41 additional lesions found in 29 patients on CEM and/or MRI

**Additional lesions**	**Number (%)**
**Number of lesions per patient**	
One	18 (62)
Two	10 (35)
Three	1 (3)
**Lesion location**	
Right breast	23 (56)
**Location within breast relative to index malignant lesion(s)**	
Same breast same quadrant	18 (44)
Same breast different quadrant	11 (27)
Contralateral breast	12 (29)
**Modality of detection**	
CEM	18 (44)^a^
MRI	35 (85)^a^
Both CEM and MRI	12 (29)
**Lesion characteristics**	
CEM (*n* = 18)	
Mass	14 (78)
Non-mass	4 (22)
MRI (*n* = 35)	
Focus	3 (9)
Mass	24 (68)
Non-mass	8 (23)

^a^Proportions do not sum to 100% due to the lesions detected by both modalities. *CEM* Contrast-enhanced mammography, *MRI* Magnetic resonance imaging

Most of the additional lesions (29/41, 71%) were in the same breast as the index cancer, of which 18/29 (62%) were in the same and 11/29 (38%) in a different quadrant. None of the additional lesions in the contralateral breast were malignant.

Six of the 41 (15%) were reported on CEM only and 23/41 (56%) on MRI only, while 12/41 lesions (29%) were seen with both tests. The detection of additional lesions by MRI was significantly higher than CEM (*p* = 0.002). The average size of the 18 additional lesions detected on CEM was 15.5 ± 12.9 mm (mean ± standard deviation), range 5–40 mm, and 13/18 (72%) were called “positive” on imaging, *i.e.*, graded NBCC ≥ 3. The majority (14/18, 78%) were masses.

Of the 35 additional lesions identified on MRI, the mean size was 11.2 ± 8.6 mm (mean ± standard deviation), range 2–35 mm, and 26/35 (74%) were called “positive”, *i.e.*, graded NBCC ≥ 3. Again, the majority (23/35, 66%) were masses. Additional lesions detected by both CEM and MRI had a mean size of 14.7 ± 11.8 mm (range 6–40 mm) for CEM and 13.5 ± 10.3 (range 6–35 mm) for MRI. Two lesions were called “positive” based on CEM alone, one on MRI alone and seven by both CEM and MRI. The commonest type of additional lesion detected on both modalities was a mass 9/12 (75%).

### Diagnostic work-up of lesions called “positive” on imaging

**Fig. 2:**
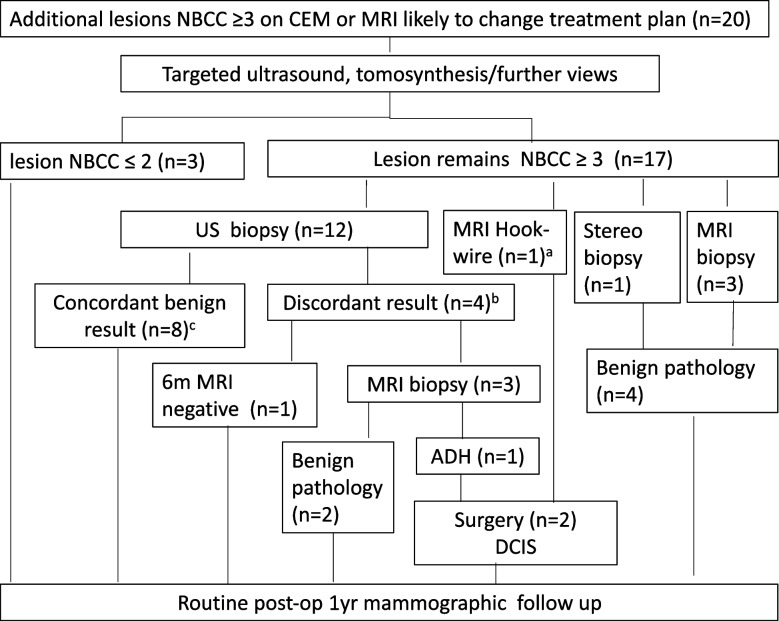
Results of assessment procedures. ^a^Additional lesion excised “en-bloc” with index lesion following preoperative MRI-guided hook-wire insertion. ^b^Discordant histopathology or biopsy marker clip position. ^c^One lesion was ADH at surgery. *ADH* Atypical ductal hyperplasia, *CEM* Contrast-enhanced mammography, *DCIS* Ductal carcinoma *in situ*, *FU* Follow-up, *MRI* Magnetic resonance imaging, *NBCC* National Breast Cancer Center, *US* Ultrasound, *Stereo* Stereotaxis, *Post-op* Post operative

Of the 32 additional lesions considered “positive” on either MRI or CEM, 20 lesions underwent further assessment (Fig. 2). Ultrasound and in some cases further mammographic views or tomosynthesis were performed and lesions that remained suspicious underwent needle biopsy using ultrasound, stereotactic, or MRI guidance. Most of the preoperative needle biopsies were performed with either 14-gauge spring-loaded needles (ultrasound guidance) or a 9-gauge vacuum-assisted core biopsy device (stereotactic guidance). Fine needle aspiration (with immediate on-site cytopathology confirmation of sampling adequacy) was performed for two lesions thought to represent intramammary lymph nodes. Marker clips were inserted following core biopsy.

Concordance between the lesion sampled using ultrasound guidance and the lesion detected on CEM or MRI was assessed by comparing marker clip position on post-biopsy mammograms with the lesion location on the CEM or MRI images. If there was concern regarding appropriate lesion sampling or radiological-pathological discordance, a repeat biopsy using MRI guidance or preoperative lesion localisation followed by diagnostic excision at the time of surgery for the known malignancy was performed.

Twelve lesions did not undergo pre-operative work-up, as this would not have changed the surgical plan (mastectomy). Four lesions were malignant, and for the remaining eight, surgical pathology showed no abnormality or benign findings.

A pathology reference standard was available for 30 of the 41 additional lesions: 24 were benign and six malignant. There were three invasive carcinomas (measuring 7 mm, 7 mm, and 15 mm) and two were DCIS (measuring 30 mm and 2 mm) (Table 2). The remaining lesion was a metastatic intramammary lymph node. Three of the additional malignant lesions were in the same quadrant of the index cancer; however, three were in another quadrant.

**Table 2:** Final diagnosis for the additional lesions as assessed by reference standards

**Additional lesions (*n* = 41)**	**Number (%)**
**Benign lesions**	35 (85%)
Non-specific benign findings^a^	28
Fibroadenoma	4
Benign intramammary lymph node	2
ADH	1
**Malignant lesions**	6 (15%)
Side	
Right	6
**Location of additional malignant lesion** **relative to index malignant lesion(s)**	
Same breast same quadrant^b^	3
Same breast different quadrant^c^	3
**Pathological features**	
**Invasive breast cancer**	3
IDC	1
IDC and DCIS	1
IDC and ILC with DCIS	1
**Histopathological Features**	
Grade 2	3
Luminal A	2
Luminal B	1
**Malignant intramammary lymph node**	1
**DCIS only**	2
High grade	1
Low grade	1

^a^Surgical or core biopsy pathology was available for 19 lesions; the remaining nine lesions were stable on follow-up for a minimum of 12 months. ^b^Two invasive malignancies, one DCIS. ^c^One invasive malignancy, one DCIS, one metastatic intramammary lymph node. *ADH* Atypical ductal hyperplasia, *DCIS* Ductal carcinoma *in situ*, *ER* Oestrogen receptor, *HER2/Neu* Human epidermal growth factor receptor 2, *IDC* Invasive duct carcinoma, *ILC* Invasive lobular carcinoma

### Comparison of CEM *versus* MRI in detection of malignant additional lesions

Comparative review of the diagnostic indices for CEM and MRI shows that although CEM detected fewer additional lesions than MRI, most (12 of 41 lesions, 29%) were false positives and only one of the six cancers was detected. In contrast, while MRI reported more false-positive lesions (20 of 41, 49%), it detected and correctly classified all six malignancies. False-positive findings on MRI resulted in 15 focused breast ultrasound exams and 15 needle biopsies. False-positive findings on CEM resulted in 7 focused ultrasounds and 8 needle biopsies.

The sensitivities of CEM and MRI were 1/6 (16.7%, 95% confidence interval 0.4–64.1%) and 6/6 (100%, 54.1–100.0%), respectively (*p* = 0.063). The positive predictive value was 1/13 (7.7%, 0.2–36.0%) for CEM and 6/26 (23.1%. 9.0–43.6%) for MRI (*p* = 0.276).

### Review of additional malignant lesions not identified by CEM

**Fig. 3:**
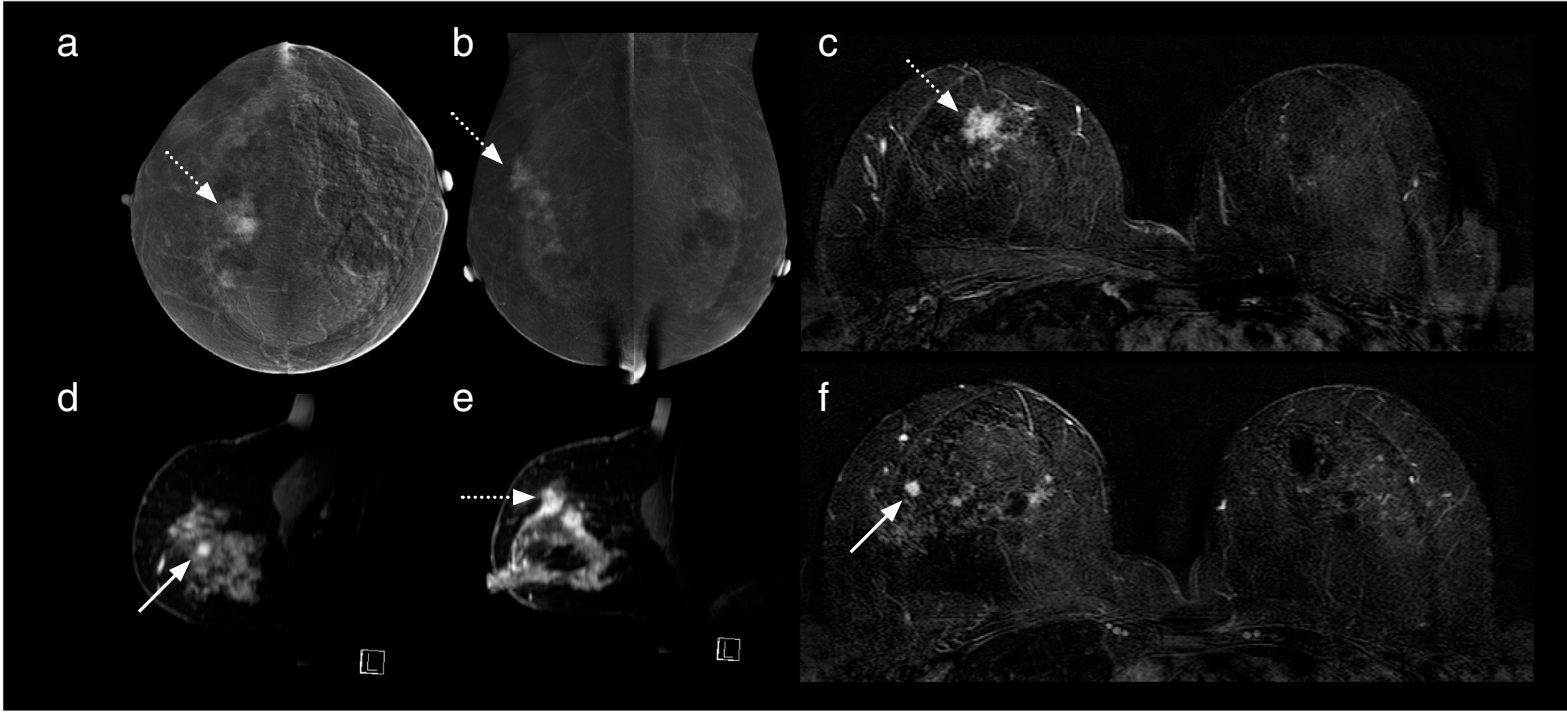
Case number 1 of Table 3. A 64-year-old woman presented with a calcified right breast mass at 12 o’clock on screening mammography, a grade 3 infiltrating ductal carcinoma. CC (**a**) and MLO (**b**) recombined CEM images show an enhancing spiculated mass (dotted arrows). Enhancing satellite foci are also visible on the MLO projection. MRI axial (**c**, **f**) and sagittal (**d**, **e**) contrast-enhanced T1-weighted subtracted images show the dominant lesion (dotted arrows) and an additional enhancing lesion solid (solid arrows) 2 cm infero-laterally. No corresponding finding was detected laterally on CEM. Mastectomy revealed a multifocal grade 3 invasive ductal carcinoma, oestrogen-, progesterone- and Her2 receptor-positive, with associated high-grade ductal carcinoma *in situ*. The additional lesion was a grade 2 invasive ductal carcinoma. *CC* Craniocaudal, *CEM* Contrast-enhanced mammography, *MLO* Mediolateral, *MRI* Magnetic resonance imaging

**Fig. 4:**
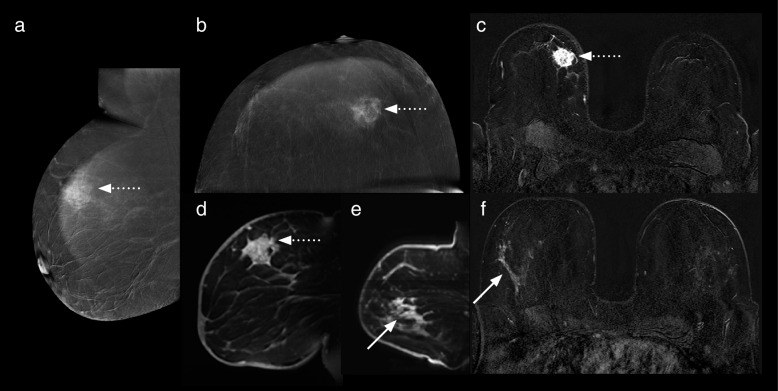
Case number 2 of Table 3. A 41-year-old woman with a palpable lump in the right breast. Recombined MLO (**a**) and CC (**b**) CEM images and first contrast-enhanced subtracted T1-weighted axial (**c**) and sagittal (**d**) MRI images. The index malignant lesion was a triple-negative, grade 3 invasive ductal carcinoma, seen here as a 15-mm ill-defined, irregularly shaped, heterogeneously enhancing mass (dotted arrows) at the 11 o’clock position in the right breast. Sagittal (**e**) and axial (**f**) post-contrast T1-weighted subtracted images from the MRI study demonstrate an additional lesion in the right lower outer quadrant, a 34-mm segmental area of non-mass enhancement (solid arrows), not visible on CEM. This additional lesion was 30-mm low-grade DCIS. The degree of enhancement of the index lesion on MRI is subjectively much greater than that shown on CEM. The noncalcified DCIS shows minimal enhancement on MRI. *CC* Craniocaudal, *CEM* Contrast-enhanced mammography, *DCIS* Ductal carcinoma *in situ*, *MLO* Mediolateral, *MRI* Magnetic resonance imaging

**Fig. 5:**
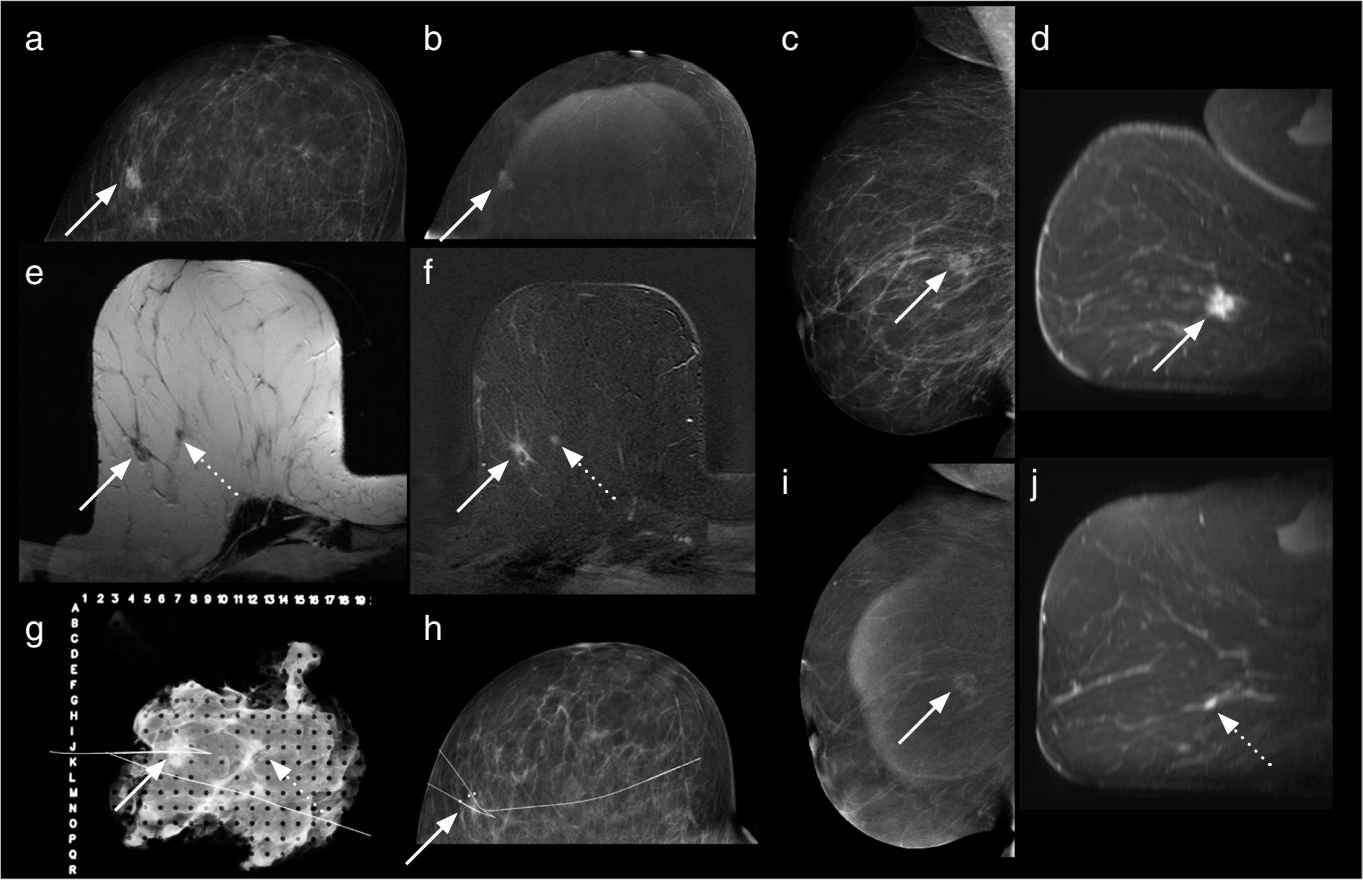
Case number 3 of Table 3. A 54-year-old patient with a screen-detected 12-mm calcified right breast mass, a grade 3 infiltrating ductal carcinoma with associated DCIS on core biopsy. CEM: (**a**) low energy and (**b**) recombined CC views as well as (**c**) low energy (**i**) recombined MLO (**i**) views show a calcified rim enhancing mass (solid arrows). MRI: axial unenhanced T1-weighted (**e**), axial T1-weighted contrast-enhanced subtracted (**f**), axial, and (**d**, **j**) sagittal images show the enhancing mass (solid arrows). The additional lesion detected on MRI was a 5-mm focus of enhancement (dotted arrow) lying 15 mm antero-mediallly to the index cancer. CC mammogram (**h**) shows the bracketing hookwires following MRI guided insertion. Grid specimen radiograph (**g**): in addition to the main calcified mass, a tiny cluster of microcalcifications (dotted arrow) is noted, not visible on the initial magnification mammograms. Final pathology: the screen-detected lesion was a 10-mm unifocal grade 3 mixed micropapillary ductal carcinoma not otherwise specified and papillary carcinoma with associated DCIS. The additional lesion was a 2-mm focus of DCIS, 20 mm away from the main lesion. *CC* Craniocaudal, *CEM* Contrast-enhanced mammography, *DCIS* Ductal carcinoma *in situ*, *MLO* Mediolateral, *MRI* Magnetic resonance imaging

**Fig. 6:**
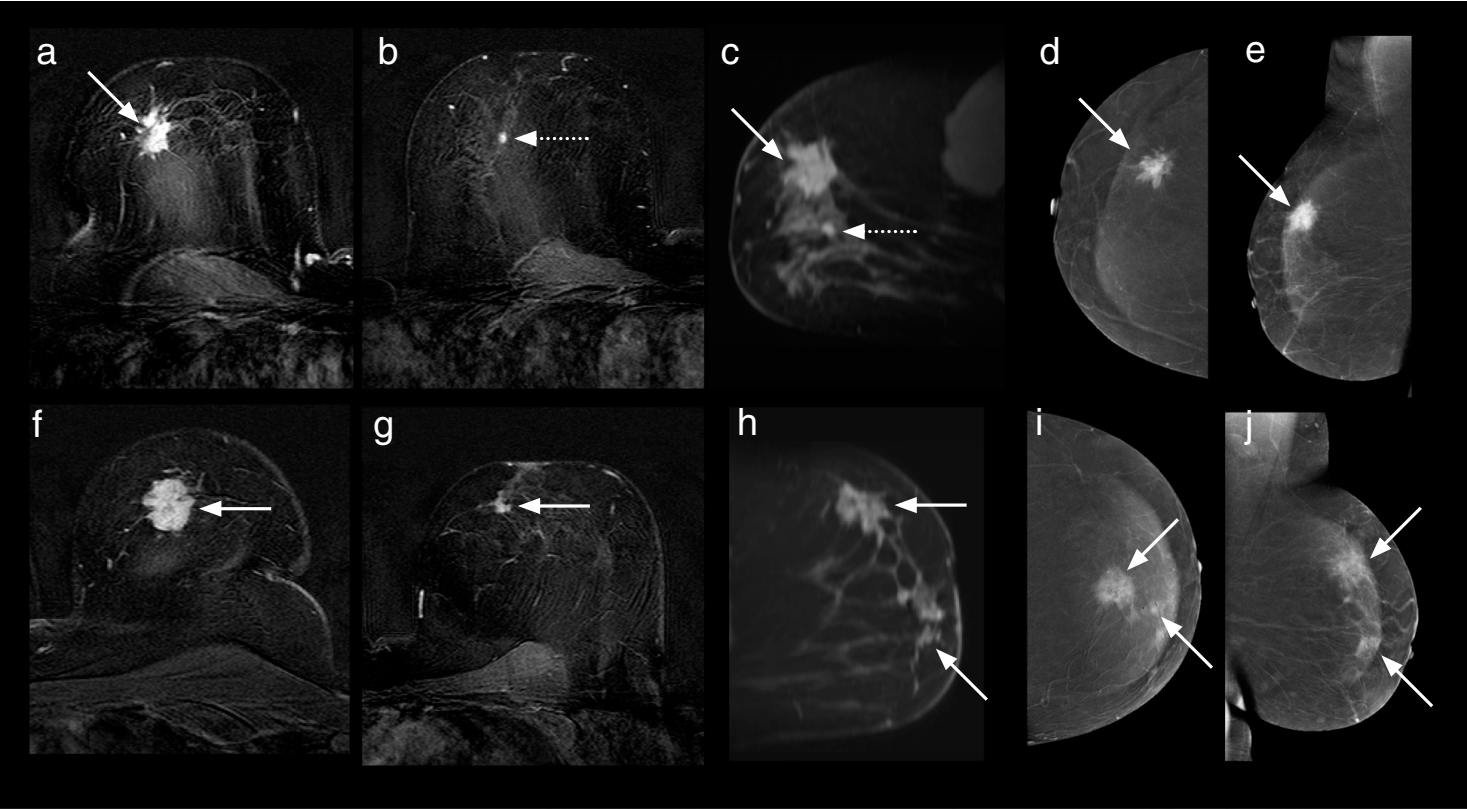
Case number 4 of Table 3. A 57-year-old patient with bilateral lesions detected on screening mammography, one on the right (grade 2 infiltrating ductal carcinoma with intermediate grade DCIS) and two on the left (both grade 2 infiltrating ductal carcinomas with DCIS). Right breast: T1-weighted post-contrast axial (**a, b**) and sagittal (**c**) MRI images;  recombined CC (**d**) and MLO (**e**) CEM images. Left breast: T1-weighted post-contrast axial (**f, g**) and sagittal (**h**) MRI images; recombined CC (**i**) and MLO (**j**) CEM images. Solid arrows point to the sites of the primary lesions initially detected on conventional imaging.  An additional right-sided lesion (dotted arrows) was noted on the MRI, 18 mm inferior to the primary lesion. This lesion was not seen on CEM - it may have been superimposed on the main lesion on the CC view and contrast “wash-out” may have occurred by the time of MLO was acquired. Confirmation of additional lesion (a 7-mm invasive ductal carcinoma grade 2 with low grade DCIS) could have changed the treatment plan. *CC* Craniocaudal, *CEM* Contrast-enhanced mammography, *DCIS* Ductal carcinoma *in situ*, *MLO* Mediolateral, *MRI* Magnetic resonance imaging

**Fig. 7:**
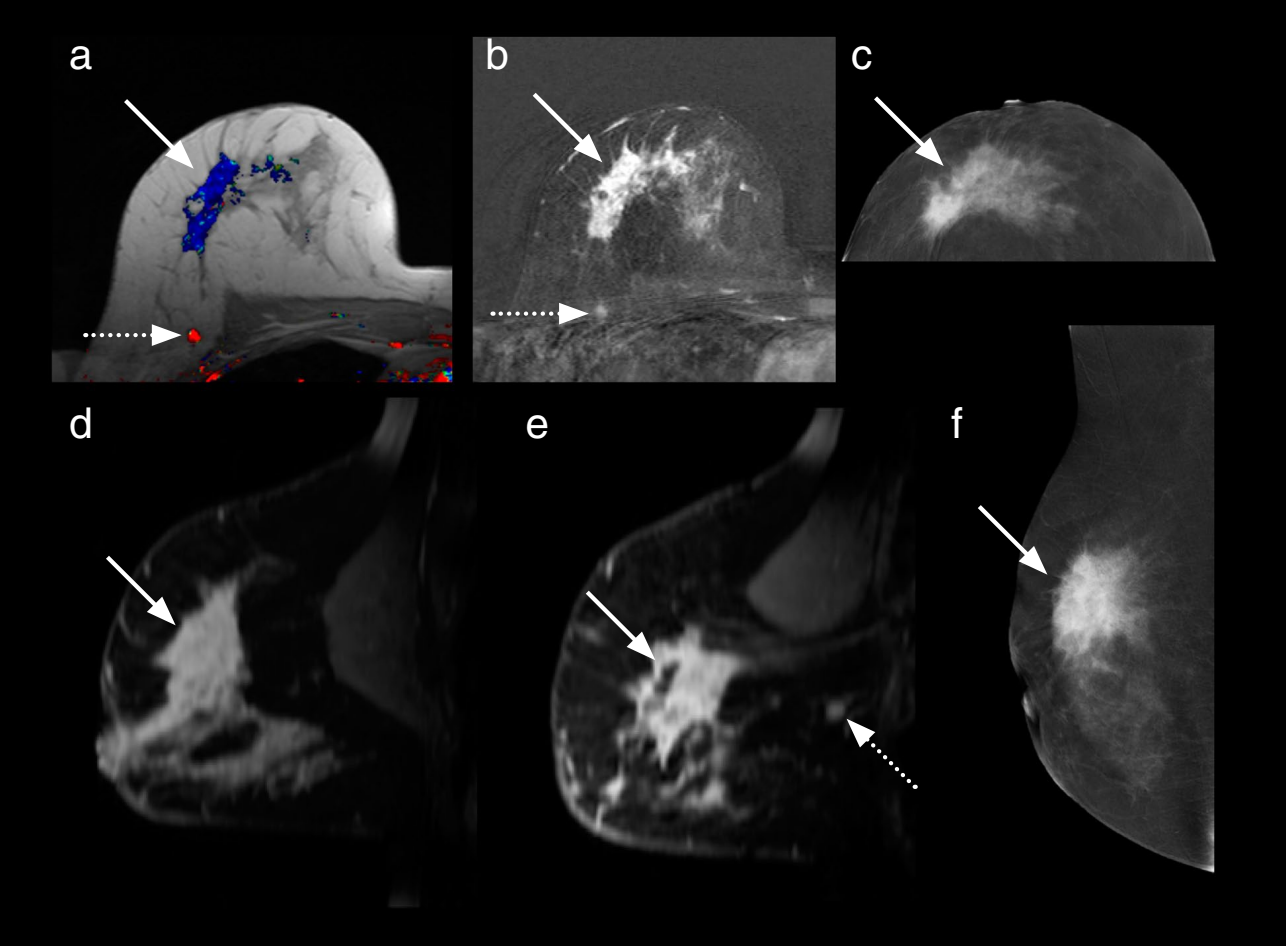
Case number 5 of Table 3. 5. A 47-year-old patient with a palpable right breast mass. Images of the right breast: MRI (**a**) axial post-contrast T1-weighted non-fat suppressed image with colour overlay, (**b**) Fat suppressed axial post-contrast (**d, e**) sagittal post-contrast T1-weighted subtraction images. CEM (**c**) recombined CC view, orientated to match the axial MRI images (**f**) recombined MLO view. CEM and MRI both demonstrated the index lesion (solid arrows), a pleomorphic grade 3 invasive lobular carcinoma. MRI showed a moderately enhancing additional 6-mm mass in the postero-superior aspect of the right breast (dotted arrows). This lesion is out of the field of view on CEM. The additional lesion was a metastatic intramammary lymph node. *CEM* Contrast-enhanced mammography, *MRI* Magnetic resonance imaging, *CC* Cranio-caudal, *MLO *Mediolateral oblique

The CEM and MRI studies of the five patients where an additional malignant lesion was not identified on CEM were reviewed to search for reasons as to why these lesions were not detected (Table 3, Figs. 3, 4, 5, 6, and 7).

**Table 3:** Features of the six additional malignant lesions

Variable	Lesion 1	Lesion 2	Lesion 3	Lesion 4	Lesion 5	Lesion 6
CEM BPE	Mild	Minimal	Minimal	Minimal	Mild	Minimal
MRI BPE	Mild	Mild	Minimal	Mild	Moderate	Minimal
MRI NBCC score	3	3	3	3	3	5
CEM finding	None	None	None	None	None^d^	Mass
CEM lesion enhancement relative to background	None	None	None	None	None	Moderate
CEM size (mm)	−	−	−	−	−	12
CEM NBCC score	−	−	−	−	−	3
MRI finding	Mass	Non-mass	Mass	Focus	Mass	Mass
MRI lesion enhancement relative to background	High	Low	Low	High	Low	High
MRI size (mm)	7	34	5	5	6	12
Histological type	IDC	DCIS	DCIS	IDC and DCIS	Metastatic lymph node (from ILC)	IDC, ILC, and DCIS
Tumour grade	2	Low	High	2	3^a^	1 and 2
Oestrogen receptor	Positive	Not done	Not done	Positive	Positive^a^	Positive
Progesterone receptor	Positive	Not done	Not done	Positive	Positive^a^	Positive
Her2/Neu	Positive	Not done	Not done	Negative	Negative^a^	Negative
Intrinsic subtype	Luminal B-like (Her2/Neu positive)	Not available	Not available	Luminal A-like	Luminal B-like^a^	Luminal A-like
Lesion size on pathology (mm)	7	30	2	7	− ^c^	15

Six additional malignant lesions were found in six different patients; all were in a breast already known to contain malignant lesion(s) detected by mammography and/or ultrasound. ^a^These values are those of the index malignant lesion. ^c^Size not recorded. ^d^Lesion out of the field of view of CEM. *BPE* Background parenchymal enhancement, *CEM* Contrast-enhanced mammography, *DCIS* Ductal carcinoma *in situ*, *HER2/Neu* Human epidermal growth factor receptor 2, *IDC* Invasive ductal carcinoma, *ILC* Invasive lobular carcinoma, *MRI* Magnetic resonance imaging

### Discussion

This study is one of a few that has performed a head-to-head comparison of the ability of CEM with that of MRI for the detection of additional foci of malignancy not previously detected on conventional imaging in a sample of women with at least one biopsy-proven invasive breast cancer. Our results suggest that MRI may be superior to CEM, as it enabled the detection of all six lesions while CEM detected just one. The fact that two of the five additional cancers missed by CEM were multicentric cancers and one of them was 30 mm in size is cause for concern (Fig. 5).

While the effect of detection of the additional cancers on the surgical plan is not evaluated in this paper, the ability of preoperative imaging to differentiate unifocal from multifocal or multicentric breast cancer has important implications, for both patient treatment and prognosis [19, 20]. Multicentric disease is often considered a contraindication to breast-conserving surgery; however, the ability of CEM or MRI to exclude multifocal/multicentric disease is a further important question as it may allow some women to avoid the morbidity of whole breast radiotherapy [21].

### Comparison of our findings with those previously reported

Variable results have been reported in previous studies comparing CEM with MRI for detection of additional malignant disease. Factors that might explain this variability include (i) differences in study design (*e.g.*, prospective *versus* retrospective, (ii) type of patients and/or lesions included (which may influence the prevalence of additional foci of malignancy in the study sample); (iii) criteria used to define an additional lesion (*e.g.*, enhancing lesions within 15 mm from an index lesion considered to be satellite components of the index or counted as additional); (iv) whether all lesions other than the index were considered additional lesions rather than just those only detected by MRI or CEM; (v) quality of radiological-pathological correlation; (vi) technical factors (such as the MRI and CEM hardware and software); and (vii) iodinated and gadolinium-based contrast material concentration and dose administered.

This study has focused solely on lesions not previously identified with conventional imaging, as we considered this would have greater clinical impact and potential to change patient treatment compared with the results of standard imaging.

Our findings are in keeping with those of Jochelson et al. [14] who performed a prospective study in 52 patients (all but one with invasive disease). These authors found 25 additional lesions, of which 16 were malignant. MRI and CEM missed the single contralateral malignant lesion. MRI depicted 15 of 16 additional cancers whereas CEM only found 9. MRI identified 11 of the 11 lesions that would have resulted in mastectomy, whereas CEM identified only 8/11 (73%). The authors concluded that MRI may be superior to CEM for the detection of additional malignant lesions.

A more recent retrospective study of 52 patients in whom 15 index lesions were DCIS [12] found 58 additional lesions, of which 11 were malignant and all detected by both CEM and MRI. However, multifocal lesions were not considered, only multicentric or contralateral lesions. Further, 42 of the 52 women had been enrolled to evaluate an additional suspicious lesion already detected on MRI, potentially increasing the prevalence of additional cancers in the sample and inflating MRI malignancy detection.

Kim et al. [11] reported a retrospective study that included 84 women with 70 additional lesions identified, of which 37 were malignant. Both CEM and MRI detected 31 of the 37 malignant additional lesions. It is uncertain why the incidence of additional cancers in their study sample was so much higher than in other studies. Some of the additional lesions may have already been detected on standard imaging rather than with CEM or MRI alone or represented satellite components of an index lesion.

In a further retrospective study of a highly selected group of 54 women already known to have multifocal or multicentric breast cancer [22], 188 lesions were found, of which 177 were cancers. No distinction was made between index and additional lesions and details regarding the method of imaging-pathology correlation were not described.

### Additional malignant lesions not detected by CEM

False-negative lesions are known to occur with both CEM and MRI but in this study were more common with CEM. However, it is important to note that in any lesion-level analysis, sensitivity is subject to extreme verification bias as only those lesions detected on imaging are assessed by the reference standard [18]. Given our small sample size, the apparent lower sensitivity of CEM *versus* MRI for detection of additional malignant lesions (17% *versus* 100%) must also be interpreted with caution, as also suggested by the lack of statistical significance, even though with a borderline *p*-value.

The five cases where MRI detected an additional malignant lesion not identified on CEM are shown in Figs. 2, 3, 4, 5, and 6. While small numbers prevented the identification of any commonality, some possible contributing factors include technical factors (*e.g.*, “rim” artefact occurring at the periphery of the breast, which can make perception of a lesion in this region difficult) [23]; updates of the recombination software released after our study could allow to eliminate this artefact [24]; lesions outside the field of view of CEM, *i.e.*, close to the chest wall (*e.g.*, Fig. 7) [25]; superimposition with index lesion (*e.g.*, Figs. 3 and 6); and degree of contrast enhancement, lower with CEM than with MRI [26]. Relatively low levels of enhancement have been noted to occur more commonly with invasive lobular and DCIS compared with invasive ductal [27] while mucinous carcinomas may not enhance at all [28].

Notably, this study was performed early in our experience with CEM and minimally enhancing lesions may have been dismissed as background enhancement. There is evidence for a learning curve for CEM interpretation [29] and further experience gained with using CEM has taught us that even minimally enhancing findings deserve further consideration, particularly in women with known breast cancer.

In addition to the degree to which it enhances, the conspicuity of a lesion may be influenced by the level of surrounding BPE. Neither the CEM or MRI studies in our patients were timed according to the menstrual cycle, nor is this done in clinical practice to avoid delays in treatment. Studies to date have not found significant variation in CEM BPE with the menstrual cycle [30] and the amount of BPE on the CEM and MRI studies in our FN group was similar (minimal-mild) on both modalities.

### False-positive lesions

The improved detection of additional malignant disease with MRI must be balanced against an overall higher false-positive rate, further imaging, needle biopsy, and potential treatment delay. In our study, MRI had almost twice as many false positives as CEM. However, this rate was lower than that observed in the study of Jochelson et al. [14], where MRI had six times more false positives than CEM. As noted by others [28, 31–34], the commonest benign entities accounting for the false-positive lesions in our study were non-specific benign breast change and fibroadenomas.

### Study limitations

Our study had some limitations. First, our initial power calculation was based on the ability of CEM and MRI to detect any additional lesion, regardless of the underlying pathology. The small number of additional otherwise occult cancers in our sample has resulted in wide confidence intervals for sensitivity and positive predictive values, which limits the strength of our conclusions, particularly in regard to the apparent higher sensitivity of MRI for additional malignancies in comparison to CEM. Second, the readers’ very limited experience with CEM could have (at least in part) been responsible for some of the CEM false negatives. Third, in any lesion-level analysis in which a lesion needs to be identified before it can be assessed, verification bias is unavoidable. As in previous studies, not all patients had bilateral mastectomies and standard tissue processing rather than large format techniques [35] was used; therefore, the true–false-negative rate is unknown. Fourth, while considerable attention was paid in finding a pathological correlate for all imaging-detected lesions, lesions located within excised breast tissue, which did not undergo image-guided biopsy with clip insertion, may not have been identified or could have been overlooked. Finally, the length of imaging follow-up in this study could also be considered relatively short but is consistent with that reported by others [11, 14].

Despite these limitations, our results do raise doubt that CEM may not be as sensitive as MRI for detection of multifocal/multicentric disease. As noted in a recent systematic review and meta-analysis [36], the paucity of data regarding the detection of CEM or MRI-only lesions means the hypothesis that CEM is non-inferior to MRI for detecting otherwise occult multifocal and multicentric disease is yet to be adequately tested. Large well-designed prospective multicentre studies are needed.

In conclusion, while the practical advantages of CEM make it a very attractive tool, our findings suggest that CEM may not be as sensitive as MRI in the preoperative setting for detecting otherwise unsuspected additional foci of malignancy that could impact the surgical treatment and final patient outcomes.

## Supplementary Information

**Additional file 1: Supplementary material.** Method of tissue processing by the pathologist.

## Data Availability

Data will be made available from the corresponding author upon receipt of a reasonable written request, including relevant Ethics Committee Approvals.

## Abbreviations

BPE: Background parenchymal enhancement
CEM: Contrast enhanced mammography
DCIS: Ductal carcinoma *in situ*
MRI: Magnetic resonance imaging
NBCC: National Breast Cancer Center
