# Supplementary material for: Contrast-enhanced mammography (CEM) versus MRI for breast cancer staging: detection of additional malignant lesions not seen on conventional imaging
Source: Eur Radiol Exp. 2023 Feb 13;7:8. doi: 10.1186/s41747-022-00318-5 (PMC9925630; doi:10.1186/s41747-022-00318-5)

ELECTRONIC SUPPLEMENTARY MATERIAL

Contrast-enhanced mammography (CEM) versus MRI for breast cancer staging: detection of additional malignant lesions not seen on conventional imaging

Method of tissue processing by the pathologist

Wide local excision specimens were received fresh and following overnight fixation in 10% neutral buffered formalin, the specimens were weighed, placed in a grid and radiographed. Coordinates of imaging lesions were identified from review of the diagram and specimen radiograph and pins inserted into the specimen through the holes in the grid based on the given coordinates. Details regarding tissue processing are given in the supplementary material.

The specimens were measured and margins inked using the surgeon's orientation sutures. The entire specimen was serially sectioned in 3-5 mm parallel slices in the sagittal plane and consecutively laid out, maintaining orientation. The lesion and margins were blocked and processed using standard processing techniques. One 4µm section was cut from each block and stained with haematoxylin and eosin (H & E).

Following formalin fixation, mastectomies were serially sectioned in 5-10 mm parallel slices in the sagittal plane, with slices either held together by overlying skin (akin to a book-end) or consecutively laid out, maintaining orientation. If a lesion was not apparent, or the pre-operative imaging/core biopsy suggested a predominantly in-situ process, slices were radiographed prior to blocking. Region(s) of interest (including marker clips) and margins were blocked then processed using standard techniques. One 4µm section was cut from each block and stained with H & E.

UMRN:	Patient ID: C E S M
Patient Name:	Date of Surgery: _____/_____/______
Pathologist name:	Signature:
Today's date: _____/_____/______	

The information provided in this red box is based on radiological findings and is intended to be an aid for the pathologist when finding the lesions in the diagram(s) above. Please use additional forms if there are >3 lesions.

Distance between edge of lesion sites - mm (please leave blank if lesions are in different breasts):	Between primary lesion and lesion 2: ___________ mm	Between primary lesion and lesion 3: ___________ mm	Between lesion 2 and lesion 3 : ___________ mm

Lesion Number	1 (primary/index lesion)	2 (additional lesion)	3 (additional lesion)
Modalities observed	MMG · US · CESM · CEMRI	MMG · US · CESM · CEMRI	MMG · US · CESM · CEMRI
Side (L or R)	L · R	L · R	L · R
Quadrant (circle)	UIQ · LIQ · UOQ · LOQ · Central	UIQ · LIQ · UOQ · LOQ · Central	UIQ · LIQ · UOQ · LOQ · Central
Clock position (1-12)	1 2 3 4 5 6 7 8 9 10 11 12	1 2 3 4 5 6 7 8 9 10 11 12	1 2 3 4 5 6 7 8 9 10 11 12
Depth	Ant third · Mid third · Post third	Ant third · Mid third · Post third	Ant third · Mid third · Post third
Size (mm).	MMG Max: SI: AP ML: · US Max: SI: AP ML: · CESM Max: SI: AP ML: · CEMRI Max: SI: AP ML:	MMG: Max: · US: Max: · CESM: Max: · CEMRI: Max:	MMG: Max: · US: Max: · CESM: Max: · CEMRI: Max:
Disease extent in each region/quadrant if measured more than one lesion in that region/quadrant	R/UIQ:AP: SI: Trans: Max:<br>R/LIQ:AP: SI: Trans: Max:<br>R/UOQ:AP: SI: Trans: Max:<br>R/LOQ:AP: SI: Trans: Max:<br>R/Central:AP: SI: Trans: Max:	L/UIQ:AP: SI: Trans: Max:<br>L/LIQ:AP: SI: Trans: Max:<br>L/UOQ:AP: SI: Trans: Max:<br>L/LOQ:AP: SI: Trans: Max:<br>L/Central:AP: SI: Trans: Max:	
Straight line distance from nipple (mm)			
Straight line distance from Pec. fascia (mm)			
BiRADS score	1 2 3 4 5 6	1 2 3 4 5 6	1 2 3 4 5 6
Tabar/Nottingan score	1 2 3 4 5	1 2 3 4 5	1 2 3 4 5
Tissue sampled?	Y · N · Result?	Y · N · Result?	Y · N · Result?

This box is for entry by the pathologist

Lesion number:	1 (primary/index lesion)		2 (additional lesion)		3 (additional lesion)	
Diagnosis pathology						
Focality (circle)	Unifocal	Multifocal	Unifocal	Multifocal	Unifocal	Multifocal
Circle more accurate measure (lesion size)	Micro	Macro	Micro	Macro	Micro	Macro
Specify max size + each dimension for accurate measure (micro/macro) (mm)	Invasive:<br>Max:<br>SI:<br>AP:<br>Trans:	Invasive +in-situ:<br>Max:<br>SI:<br>AP:<br>Trans:	Invasive:<br>Max:	Invasive +in-situ:<br>Max:	Invasive:<br>Max:	Invasive +in-situ:<br>Max:
Specify disease extent in each region/quadrant if measured more than one lesion in that region/quadrant (mm).	R/UIQ:AP: SI: Trans: Max:<br>R/LIQ:AP: SI: Trans: Max:<br>R/UOQ:AP: SI: Trans: Max:<br>R/LOQ:AP: SI: Trans: Max:<br>R/Central:AP: SI: Trans: Max:		L/UIQ:AP: SI: Trans: Max:<br>L/LIQ:AP: SI: Trans: Max:<br>L/UOQ:AP: SI: Trans: Max:<br>L/LOQ:AP: SI: Trans: Max:<br>L/Central:AP: SI: Trans: Max:			
Straight line nipple distance (mm)						

Distance between edge of lesion sites - mm (please leave blank if lesions are in different breasts):	Between primary lesion and lesion 2: ___________ mm	Between primary lesion and lesion 3: ___________ mm	Between lesion 2 and lesion 3: ___________ mm

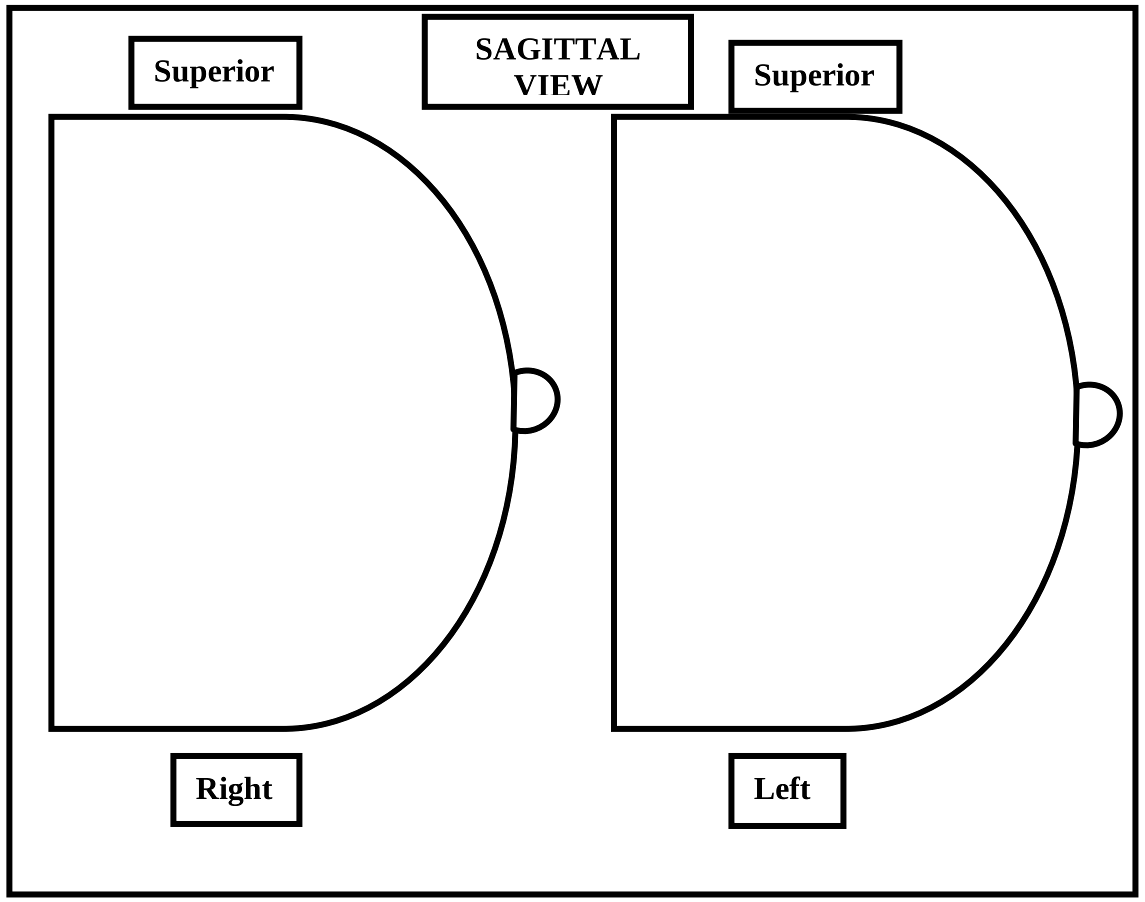

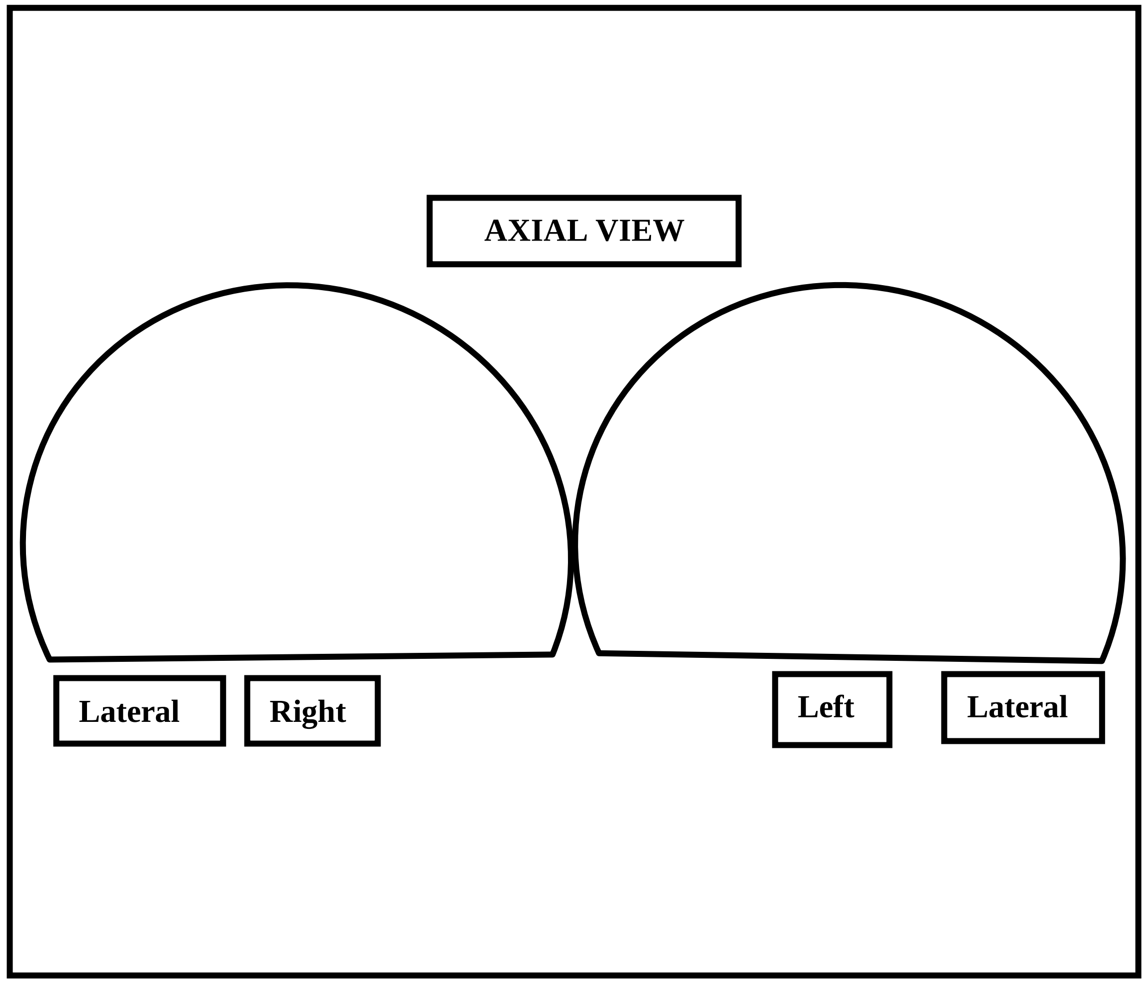

Supplement: Supplementary file 1 — Additional file 1: Supplementary material. Method of tissue processing by the pathologist. [file 41747_2022_318_MOESM1_ESM.pdf]
